# A case of choroidal metastasis from renal cell carcinoma significantly reduced by radiotherapy

**DOI:** 10.1002/iju5.12819

**Published:** 2024-12-06

**Authors:** Koichiro Kanazawa, Shinnosuke Oishi, Akihiko Sakamoto, Kuniaki Tanabe, Kazutaka Sugiyama, Akihiko Matsumoto, Akari Arakawa, Hiromi Matsunaga, Takafumi Harada, Haruki Kume

**Affiliations:** ^1^ Department of Urology Yaizu City Hospital Yaizu Shizuoka Japan; ^2^ Department of Ophthalmology Yaizu City Hospital Yaizu Shizuoka Japan; ^3^ Yaizu Kogawa Ganka Yaizu Shizuoka Japan; ^4^ Department of Urology The University of Tokyo Bunkyo City Tokyo Japan

**Keywords:** choroidal metastasis, radiotherapy, renal cell carcinoma

## Abstract

**Introduction:**

Choroidal metastasis from renal cell carcinoma is relatively rare and unresponsive to systemic treatment.

**Case presentation:**

A man in his eighties with left renal cell carcinoma and pulmonary metastasis developed visual impairment in the left eye during primary treatment with ipilimumab and nivolumab followed by secondary treatment with cabozantinib. Consultation with an ophthalmologist revealed choroidal metastasis, which was subsequently treated with local radiotherapy (3 Gy × 10 Fr), resulting in a significant reduction in the metastatic lesions.

**Conclusion:**

If visual impairment arises while treating renal cell carcinoma, it is essential to consider the possibility of intraocular metastasis. In terms of treatment, local therapies such as radiotherapy should be taken into consideration.

**Keynote message**

Radiotherapy was administered for choroidal metastasis from RCC, which led to a reduction in metastatic lesions.

**Abbreviation & Acronym**
RCC: renal cell carcinoma

## Case presentation

**Fig. 1:**
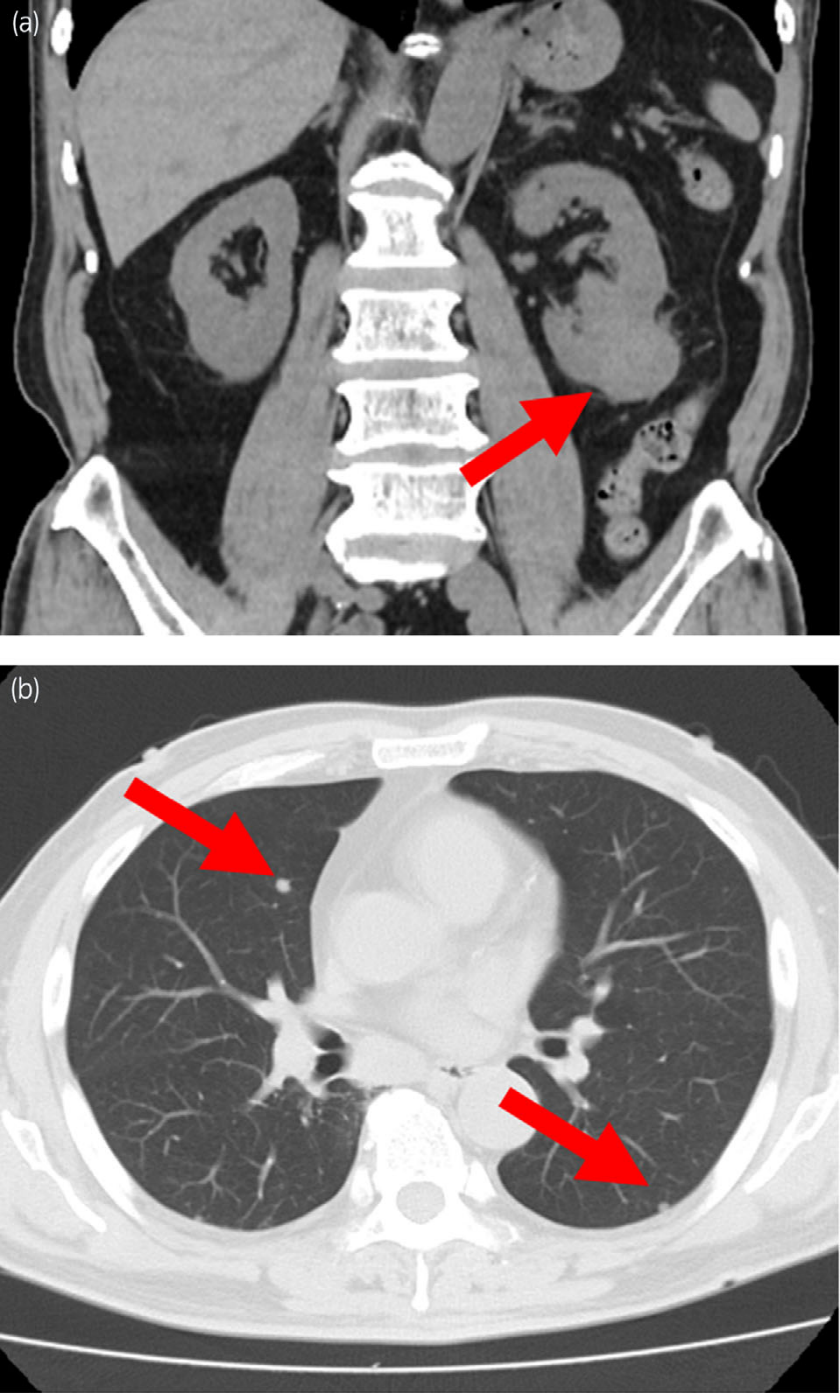
(a) Non‐contrast coronal computed tomography (CT) image showing a tumor measuring 34 mm in diameter in the left upper pole of the kidney. (b) Axial CT image showing multiple lung metastases in both lung fields.

**Fig. 2:**
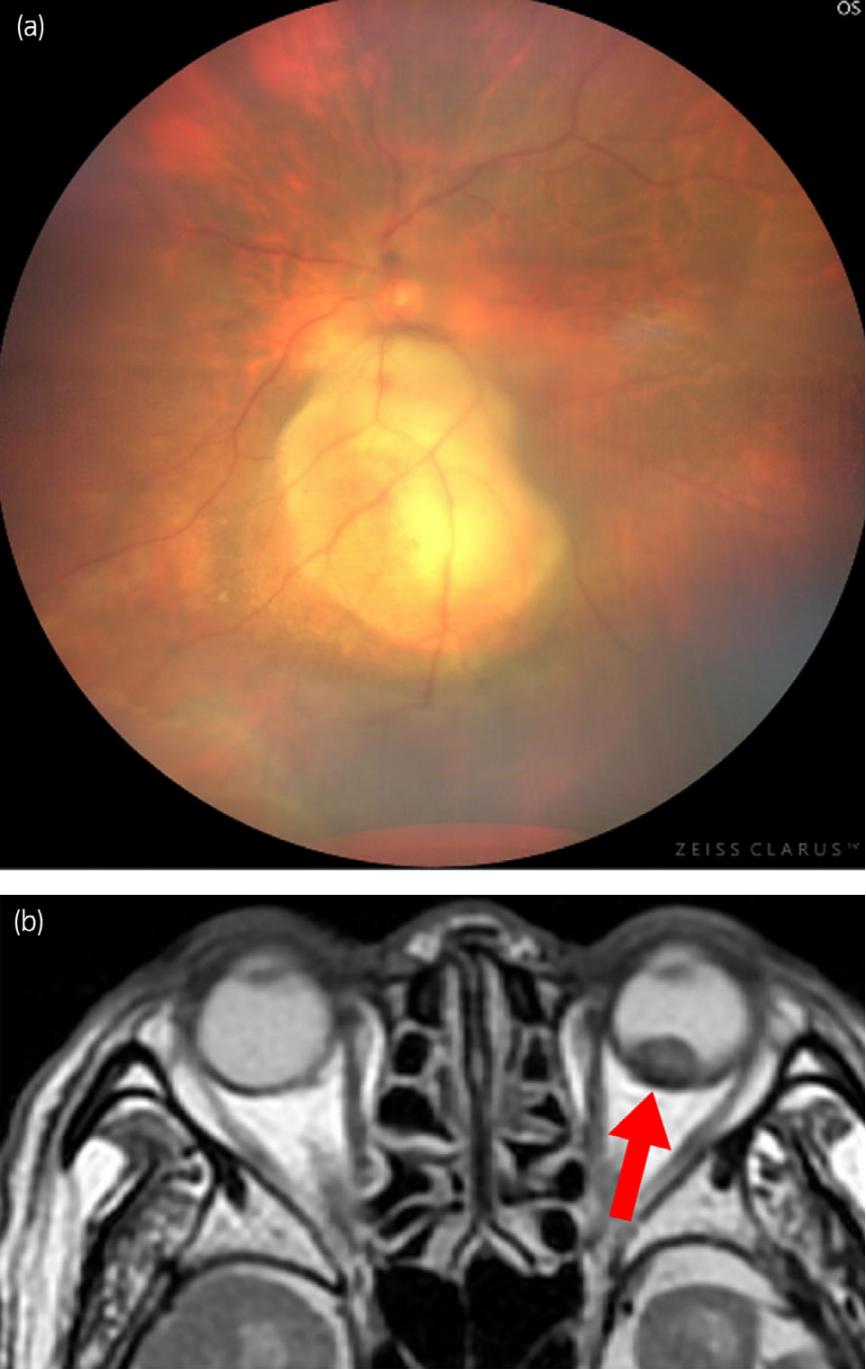
(a) Fundus examination revealing serous retinal detachment and a tumor in the choroid. (b) T2‐weighted axial magnetic resonance imaging (MRI) revealing a hypointense mass within the left eye measuring 10 mm in diameter.

**Fig. 3:**
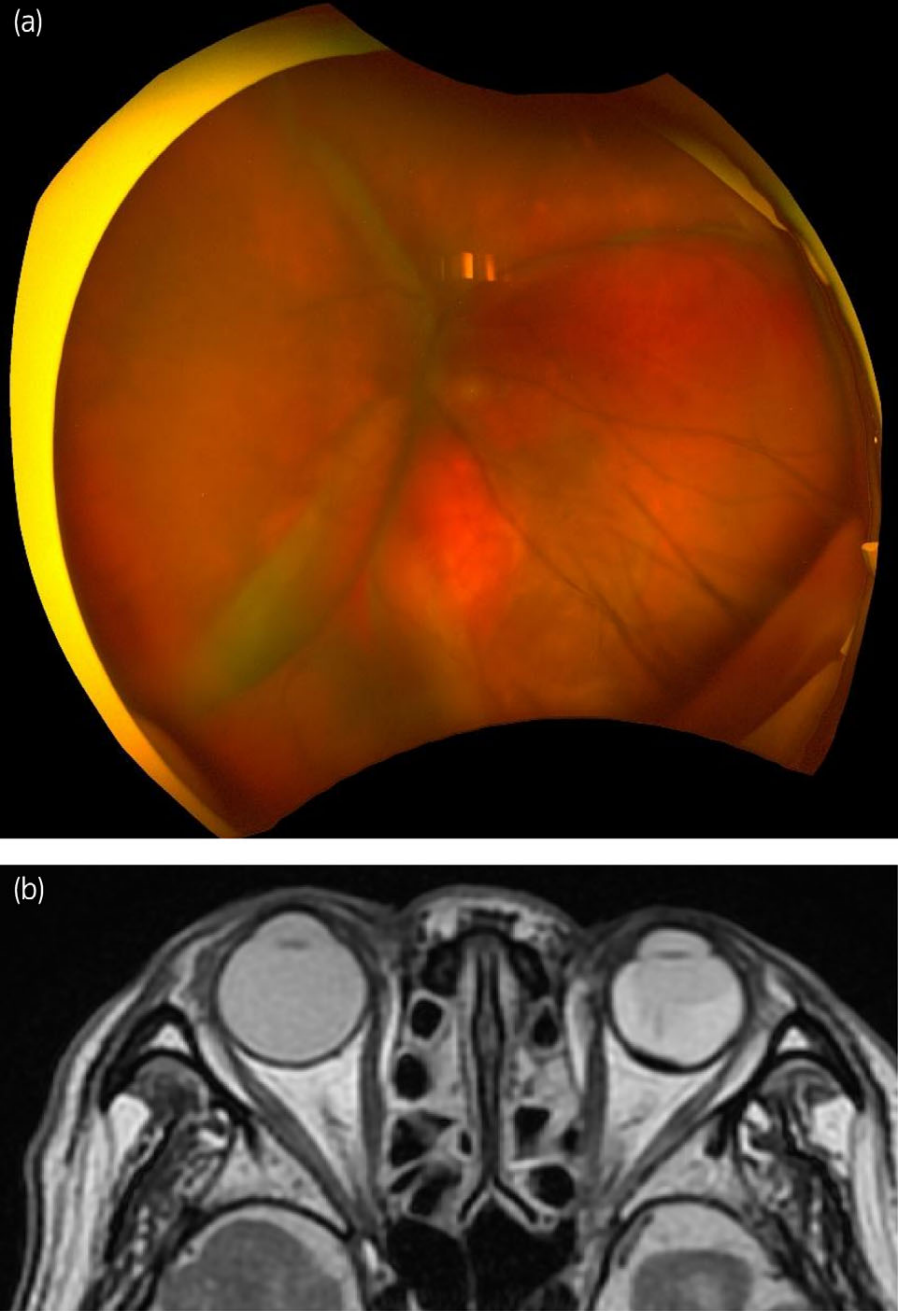
(a) Fundus examination after treatment found no tumor but the retinal detachment persisted. (b) T2‐weighted axial magnetic resonance imaging (MRI) showing that the choroidal metastasis treated with radiotherapy at 30 Gy gradually disappeared over 15 months.

A man in his eighties with a medical history of hypertension and hypothyroidism was referred to our urology department because of renal impairment identified during a health checkup. Laboratory data revealed elevated serum creatinine levels (1.5 mg/dL). His hemoglobin was 15.1 g/dL, corrected calcium was 9.6 mg/dL, platelet count was 20.8 × 10^4^/μL, and neutrophils were 3800/μL, all of which were within the normal range. Non‐contrast computed tomography (CT) revealed a mass measuring 34 mm in diameter at the lower pole of the left kidney (Fig. 1a), along with multiple lung metastases (Fig. 1b). The patient was diagnosed with left RCC with multiple pulmonary metastases (IMDC risk classification: intermediate risk). The patient underwent laparoscopic left nephrectomy (pathological diagnosis: clear cell RCC, pT3a). Two months after surgery, a combination of ipilimumab and nivolumab was administered for four cycles, followed by five cycles of nivolumab monotherapy. However, owing to the observed progression of lung metastases, the treatment was switched to cabozantinib, resulting in a reduction in lung metastases. Two years after surgery, the patient presented with floaters and upper visual field defects in the left eye. In the course of an ophthalmological consultation, fundus examination revealed a pale‐yellow tumor in the choroid and serous retinal detachment in the left eye (Fig. 2a). Differential diagnoses included hemangioma, neovascularization, and posterior scleritis in benign cases, and metastasis from cancer, malignant melanoma, osteoma, and malignant lymphoma in malignant cases. T2‐weighted magnetic resonance imaging (MRI) revealed a hypointense mass within the left eye, which was suspected to be a choroidal metastasis of the RCC. The maximum diameter was 10 mm (Fig. 2b). Temsirolimus treatment was initiated; however, even after four cycles, choroidal metastasis tended to increase. Therefore, stereotactic radiotherapy (30 Gy in 10 fractions) was administered for the choroidal metastasis, resulting in lesion reduction. Eight months after radiotherapy, the medication was changed to pazopanib due to the worsening of lung metastasis. The choroidal metastasis gradually disappeared over 15 months, although the preexisting retinal detachment remained (Fig. 3a,b).

## Discussion

Ocular metastases from malignant tumors were first reported in 1872 and are relatively rare.^1^ Ocular metastatic tumors have been observed in 12% (28 of 230) of autopsies in cancer patients.^2^ The primary tumors causing choroidal metastasis are lung cancer (37%), breast cancer (33%), kidney cancer (13%), gastrointestinal cancer (7%), thyroid cancer (7%), and parathyroid and prostate cancer (2%).^3^ In the epidemiology of ocular metastasis from renal cancer, the average age is 58.8 years and the male‐to‐female ratio is 3.4:1.^4^ Self‐reported symptoms include visual disturbances in 55%–70% of cases, which are often associated with lesions in the macular or peripapillary regions or serous retinal detachment. Visual acuity reduction has been observed in 15% of cases, visual field defects in 12%, and pain in 6%–12%. Approximately 15%–20% of cases are asymptomatic.^5^

Ancillary diagnostic tests for choroidal metastasis include color photography, autofluorescence imaging, angiography, optical coherence tomography, CT, and MRI. Choroidal metastases generally appear as creamy white or pale‐yellow masses. In rare cases, orange‐colored metastases from RCC may be observed.^6^ Ultrasound morphology was dome‐shaped, plateau‐shaped, or mushroom‐shaped. Choroidal metastases of RCC are typically dome‐shaped.^3^ MRI can detect choroidal metastases that appear isointense on T1‐weighted images and hypointense on T2‐weighted images.^6^ The tumor in the present case appeared pale‐yellow and dome‐shaped on fundus examination. MRI findings revealed a hypointense mass in T2‐weighted images. Upon ophthalmological consultation, these findings in conjunction with a known history of primary metastatic RCC were considered consistent with choroidal metastasis.

Radiotherapy of the eyeball is a common choice for treating metastatic choroidal tumors. Most patients are treated with doses ranging from 30 to 40 Gy.^6^ A prospective study by Wiegel *et al*. reported improvements in visual acuity in 36% (18 of 50) of eyes with choroidal tumors, and maintenance of vision in 50% (25 of 50).^7^ Another report indicated that visual acuity improved in 13 of 15 patients (84.6%).^8^ In all the above reports, acute adverse events such as skin inflammation, conjunctivitis, and dry eye disease were observed, but no adverse events with a radiation toxicity grading (RTOG) symptom classification of grade 3 or higher were reported.

In our case, the patient noticed the onset of floaters and later developed choroidal metastasis. Despite systemic treatment, the tumor continued to grow as the disease progressed.

There were concerns regarding potential blindness and direct intracranial invasion; therefore, stereotactic radiotherapy (30 Gy in 10 fractions) was administered in addition to systemic treatment. The combination of radiotherapy resulted in tumor reduction. However, due to age‐related cataracts and concomitant serous retinal detachment, full visual recovery was not achieved; however, blindness was prevented. In cases requiring a shorter period of radiotherapy prior to initiating a next round of chemotherapy or in which the patient is in poor condition, a palliative hypofractionated schedule (20 Gy in five fractions) has been reported for choroidal metastases.^9^ In this study, 80% (56 of 70) patients were stable or improved at a median of 13 months. No acute toxicities occurred in 89% (49 of 55) of the patients. Optic neuropathy developed in seven eyes (grade 4 in one eye and grade 3 or lower in the others). These results are acceptable compared with the previously standardized radiotherapy doses of 30–40 Gy for choroidal metastases. When a patient requires palliative treatment for any of various reasons, short‐fractionated radiotherapy may be worth considering.

## Conclusion

We reported a case of choroidal metastasis from RCC. When visual impairment is observed, a differential diagnosis of ocular metastasis should be considered. This patient was unresponsive to systemic treatment; however, we achieved a reduction in the number of metastatic lesions with radiotherapy.

## Author contributions

Koichiro Kanazawa: Conceptualization; data curation; writing – original draft; writing – review and editing. Shinnosuke Oishi: Data curation. Akihiko Sakamoto: Data curation. Kuniaki Tanabe: Data curation. Kazutaka Sugiyama: Data curation. Akihiko Matsumoto: Writing – review and editing; supervision. Akari Arakawa: Data curation. Hiromi Matsunaga: Investigation; supervision. Takafumi Harada: Supervision; investigation. Haruki Kume: Writing – review and editing; supervision.

## Conflict of interest

The authors declare no conflict of interest.

## Approval of the research protocol by an Institutional Reviewer Board

Not applicable.

## Informed consent

Not applicable.

## Registry and the Registration No. of the study/trial

Not applicable.
